# Cannabinoid receptor CB2 ablation protects against TAU induced neurodegeneration

**DOI:** 10.1186/s40478-021-01196-5

**Published:** 2021-05-17

**Authors:** M. Galán-Ganga, C. Rodríguez-Cueto, J. Merchán-Rubira, F. Hernández, J. Ávila, M. Posada-Ayala, J. L. Lanciego, E. Luengo, M. G. Lopez, A. Rábano, J. Fernández-Ruiz, I. Lastres-Becker

**Affiliations:** 1 Department of Biochemistry, School of Medicine, Universidad Autónoma de Madrid (UAM), Spain. Instituto de Investigación Sanitaria La Paz (IdiPaz), Instituto de Investigaciones Biomédicas “Alberto Sols” UAM-CSIC, C/ Arturo Duperier, 4, 28029 Madrid, Spain; 2 Instituto Universitario de Investigación en Neuroquímica, Departamento de Bioquímica Y Biología Molecular, Facultad de Medicina, Universidad Complutense, and Instituto Ramón Y Cajal de Investigación Sanitaria (IRYCIS), Madrid, Spain; 3 Centro de Biología Molecular Severo Ochoa, CSIC/UAM, Universidad Autónoma de Madrid, Cantoblanco, Madrid, Spain; 4 Faculty of Experimental Sciences, Universidad Francisco de Vitoria, Madrid, Spain; 5 Department of Neurosciences, Center for Applied Medical Research (CIMA), University of Navarra, Pio XII Ave 55, Edificio CIMA, 31008 Pamplona, Navarra Spain; 6 Instituto Teófilo Hernando y Departamento de Farmacología Y Terapéutica, Facultad de Medicina, Universidad Autónoma de Madrid, 28029 Madrid, Spain; 7 Department of Neuropathology and Tissue Bank, Unidad de Investigación Proyecto Alzheimer, Fundación CIEN, Instituto de Salud Carlos III, Madrid, Spain; 8 Centro de Investigación Biomédica en Red de Enfermedades Neurodegenerativas (CIBERNED), Madrid, Spain

**Keywords:** TAU, Cannabinoid receptor, CB_2_, Alzheimer’s disease, Neurodegeneration, Neuroinflammation

## Abstract

Tauopathies are a group of neurodegenerative diseases characterized by the alteration/aggregation of TAU protein, for which there is still no effective treatment. Therefore, new pharmacological targets are being sought, such as elements of the endocannabinoid system (ECS). We analysed the occurrence of changes in the ECS in tauopathies and their implication in the pathogenesis. By integrating gene expression analysis, immunofluorescence, genetic and adeno-associated virus expressing TAU mouse models, we found a TAU-dependent increase in CB_2_ receptor expression in hippocampal neurons, that occurs as an early event in the pathology and was maintained until late stages. These changes were accompanied by alterations in the endocannabinoid metabolism. Remarkably, CB_2_ ablation in mice protects from neurodegeneration induced by hTAU^P301L^ overexpression, corroborated at the level of cognitive behaviour, synaptic plasticity, and aggregates of insoluble TAU. At the level of neuroinflammation, the absence of CB_2_ did not produce significant changes in concordance with a possible neuronal location rather than its classic glial expression in these models. These findings were corroborated in post-mortem samples of patients with Alzheimer’s disease, the most common tauopathy. Our results show that neurons with accumulated TAU induce the expression of the CB_2_ receptor, which enhances neurodegeneration. These results are important for our understanding of disease mechanisms, providing a novel therapeutic strategy to be investigated in tauopathies.

**Supplementary Information:**

The online version contains supplementary material available at 10.1186/s40478-021-01196-5.

## Background

TAU protein is the major component of the intracellular filamentous deposits that characterize several neurodegenerative diseases termed tauopathies, which include Alzheimer's disease (AD), frontotemporal lobar degeneration (FTLD-TAU), progressive supranuclear palsy, corticobasal degeneration, among others [42]. In general, alterations in synaptic plasticity, cell death, proteinopathy, and neuroinflammation are common features in tauopathies. Pathogenic mutations in the TAU-encoding MAPT gene underlying familial frontotemporal dementia (FTD), such as TAU^P301L^ or TAU^P301S^, have supported the generation of multiple mouse models that recapitulate pathological and/or behavioural aspects of this disease. These TAU mutations reduce the ability of the protein to interact with microtubules and increase its propensity to assemble into abnormal filaments. On the other hand, there are other tauopathies where the dysregulation of TAU protein derives from different posttranslational changes, e.g. hyperphosphorylation, inducing the formation of neurofibrillary tangles, harmful to the neuron, as in the case of AD. Despite the huge efforts made to find a therapy, there is still no effective treatment for tauopathies, so finding neuroprotective treatments for these incapacitating diseases have become a priority. Actually, the failure of different clinical trials with drugs targeting TAU protein has pointed out the need for finding innovative therapeutic approaches [18].

Over the last decades, the endocannabinoid system (ECS), in particular some of its receptors and hydrolysing enzymes, has emerged as a new promising target for neurodegenerative diseases [10, 29, 57]. Recent studies have shown that the ECS modulates synaptic plasticity, as well as the neuronal homeostasis, integrity, and survival, which may be of great interest in disorders involving neurodegeneration and neuroinflammation [1]. The ECS is formed by two G protein-coupled receptors named cannabinoid receptor type-1 (CB_1_) and type-2 (CB_2_), their ligands and the enzymes responsible for their synthesis and degradation. Endocannabinoids (ECs) are amides, esters, and ethers of long-chain polyunsaturated fatty acids that act as lipid mediators [9], with *N*-arachidonoyl ethanolamine (AEA, or anandamide) and 2-arachidonoylglycerol (2-AG) being the main endogenous ligands of cannabinoid receptors. AEA is synthesized from *N*-arachidonoyl phosphatidylethanolamine (NAPE) due to the action of a specific NAPE-phospholipase D (NAPE-PLD), whereas diacylglycerol lipases α and β (DAGL α/β) are responsible for the synthesis of 2-AG from diacylglycerol (DAG) substrates (Fig. 3a). Intracellular degradation of both ECs is triggered by many different enzymes, with fatty acid amide hydrolase (FAAH), which acts on 2-AG and, in particular, on AEA, and monoacylglycerol lipase (MAGL), which act specifically on 2-AG, as the most active [9] (Fig. 3a). Also, ECs may serve as precursors for the synthesis of novel signaling lipids (e.g. prostamides, prostaglandin-glyceryl esters) when they behave as substrates for arachidonate-related enzymes as cyclooxygenase-2 (COX-2) and 15-lipoxygenase (15-LOX) (Fig. 3a).

Cannabinoid receptors have been identified as possible therapeutic targets against different neurodegenerative disorders with very positive results in preclinical studies [37, 38, 40, 46, 60]. This has included the activation of both CB_1_ and CB_2_ receptors, although the overactivation of CB_1_ receptors can lead to detrimental psychotropic effects. Moreover, it has been described a dual neuroprotective/neurotoxic profile of cannabinoid drugs, for example with Δ9‐tetrahydrocannabinol [15, 61]. By contrast, activation of CB_2_ receptors does not appear to produce these serious adverse effects, although most of compounds with CB_2_ affinity also share affinity for CB_1_ subtype [63]. Therefore, selective modulation of CB_2_ receptors has aroused great interest to exert those beneficial effects without alterations in mood or perception [13].

CB_2_ cannabinoid receptors have been traditionally found in higher levels in cells from the immune system and microglia [30], where they participate in the modulation of inflammation [16, 48]. However, recent publications have pointed their expression in neurons of the central nervous system, despite the problems of specificity shown by the different antibodies developed so far (review [8]). Using additional methodological tools (e.g. fluorescence in situ hybridization; proximity ligand assays), a neuronal location for the CB_2_ receptor has been much more proved in some specific neuronal subpopulations [64]. This recent presence in neurons has facilitated the involvement of CB_2_ receptors in the modulation of different neurobiological processes, for example, neuroplasticity and memory [44], processes that are also impaired in tauopathies. Interestingly, it has been found that CB_2_ receptors are selectively overexpressed in cells associated with amyloid-β (Aβ) enriched neuritic plaques in AD samples from postmortem human brains [11]. Nevertheless, the specific effects that TAU protein dysregulation may have on CB_2_ expression and activities have not been well established yet.

In this work, we focused on the relation between dysregulated TAU and CB_2_ receptors in different tauopathy mouse models. We also wanted to explore this relation in post-mortem human brain samples of tauopathies, in particular from TAU-dependent FTD, but samples from this specific pathology are difficult to obtain due to its low incidence, so we have to concentrate this study in AD, whose samples are easier to be collected. First, we analysed whether TAU overexpression modulates CB_2_ receptor expression in early and late-stage tauopathy mouse models and determined whether this effect is TAU-dependent. Secondly, we studied whether hTAU^P301L^ overexpression could alter ECS signalling. Due to the possible role that CB_2_ may have in memory [65] and the potential of this receptor and other elements of the ECS to exert neuroprotection by blocking of microglial activation [56], we elucidated whether the induction of CB_2_ receptors and their activation could have a positive or negative effect on the progression of tauopathy-associated neurodegeneration. For this purpose, we analysed the involvement of the CB_2_ receptor in the neurodegeneration induced by hTAU^P301L^ using CB_2_-deficient mice. We compared the cognitive impairment induced by hTAU^P301L^ between wild type (*Cnr2*^+*/*+^) and CB_2_-knockout (*Cnr2*^*−/−*^) mice and the results were correlated with the phosphorylation and aggregation status of TAU. Furthermore, we studied the implication of CB_2_ in the neuroinflammatory process in this model, given the classic role assigned to this receptor concerning the control of glial activation and reactivity [48]. Finally, we wanted to translate the issue to the human pathology scenario by determining whether CB_2_ levels could be altered in postmortem samples from the hippocampus of AD patients, the most common tauopathy.

Overall, our work offers timely insight into the role of CB_2_ receptor in tauopathies and highlights pharmacological modulation of CB_2_ receptor as a potential therapy in TAU-associated diseases.

## Methods

### Animals and stereotaxic injections

7 and 12-month-old transgenic mice overexpressing hTAU^P301S^ protein (B6;C3-Tg(Prnp-MAPT*P301S)PS19Vle/J, The Jackson Laboratory) and 10-month-old TAU-knockout mice (B6.129X1-Mapttm1Hnd/J, The Jackson Laboratory) were used. Tg- hTAU^P301S^ carried a mutant (P301S) human microtubule-associated protein tau (MAPT) gene driven by the mouse prion-protein promoter (Prnp). These animals showed progressively accumulated TAU in association with striking neuron loss as well as hippocampal and entorhinal cortical atrophy by 9–12 months of age [69]. Each experimental group comprised 4–5 mice. Regarding the experiments with CB_2_-knockout mice [45], each experimental group comprised 22–30 wild type mice (C57BL/6 J, The Jackson Laboratory) and 19–21 CB_2_-knockout mice of 6 months of age. Recombinant adeno-associated viral vectors of serotype 6, which express hTAU^P301L^ under control of the human synapsin 1 gene promoter (AAV-hTAU^P301L^), were injected in the right hippocampus (ipsilateral side) as described elsewhere [21]. In brief, 2 μL of viral suspension containing 2.1 × 10E11 GC/ml were injected at the stereotaxic coordinates − 1.94 mm posterior, − 1.4 mm lateral, and − 1.8 mm ventral relative to bregma. Three weeks after injection, mice were sacrificed, and the left side (contralateral side) was used as a control. All experiments were performed in a P2 biosafety facility and by certified researchers according to regional, national, and European regulations concerning animal welfare and animal experimentation, and were authorized by the Ethics Committee for Research of the Universidad Autónoma de Madrid and the Comunidad Autónoma de Madrid, Spain, with Ref PROEX 279/14, following institutional, Spanish and European guidelines (Boletín Oficial del Estado (BOE) of 18 March 1988 and 86/609/EEC, 2003/65/EC European Council Directives).

### Randomization and blinding

Animals were randomized for treatment. Data collection and evaluation of all experiments were performed blindly of the group identity. The data and statistical analysis with the recommendations on experimental design and analysis in pharmacology [23].

### Analysis of mRNA levels by quantitative real-time PCR

Total RNA extraction, reverse transcription, and quantitative polymerase chain reaction (qRT-PCR) was done as detailed in previous articles [21, 39]. Briefly, total RNA was extracted using TRIzol® reagent according to the manufacturer’s instructions (Invitrogen). One microgram of RNA from each experimental condition was treated with DNase (Invitrogen) and reverse-transcribed using 11 µl high capacity RNA-to-cDNA Master Mix (Applied Biosystem). Primer sequences are shown in Additional file 4: Table S1. Data analysis was based on the ΔΔCT method with normalization of the raw data to housekeeping genes (Applied Biosystems). All PCRs were performed in triplicates.

### Quantification of EC levels by LC–MS

In brief, frozen ipsilateral and contralateral hippocampi from wild type mice injected unilaterally with AAV-hTAU^P301L^ were weighed and homogenized in methanol containing 5 μl of N-arachidonoyl ethanolamine-d8 (AEA-d8), 2-arachidonoylglycerol-d8 (2-AG-d8) and 1-arachidonoylglycerol-d8 (1-AG-d8) (Cayman Chemical) as internals standards. Lipids were extracted with chloroform: H_2_O 0.1% Formic Acid 2:1. The organic phase was collected and dried in SpeedVac at 60 °C and samples were reconstituted in methanol for Liquid Chromatography-Mass Spectrometry (LC–MS) analyses. Samples were analysed by LC–MS using an Acquity H class (UPLC H-Class, Waters) online QTrap 4500 system (Sciex), and Acquity HSS T3 column (1.2 × 100 mm and 1.8 µm), as described elsewhere [53]. Briefly, a total of 5 μl of the stock solution containing extracted EC was injected and separated using precolumn and column with mobile phase A (0.1% formic acid in double distilled water Milli-Q, Millipore System) and mobile phase B (acetonitrile, Merck). The mass spectrometer was operated in positive ionization mode. For the quantification of EC, two calibration curves were realized using commercial EC standards. Individual signals were normalized based on total weight to account for sample variability and normalized peak areas for internal standard (MultiQuant software, Sciex).

### Behavioural test

The novel object recognition (NOR) test was used to assess recognition memory and was performed as described [43]. Briefly, the first day mice were placed in the empty open field and were allowed to explore the open field for 5 min. as short habituation. 24 h after, mice were placed in the open field with two identical objects for 7 min, as familiarization session. Finally, the third day mice were placed in the open field test where one of the objects has been replaced by a novel object for 7 min, as test session. The amount of time spent exploring the novel (TN) or familiar (TF) object was recorded and the differences were represented as Discrimination Index (DI). DI allows discrimination between the novel and familiar objects: [DI = (TN − TF)/(TN + TF)]. Each experimental group comprised 8–15 animals and the test was performed once two days before sacrifice.

### Immunofluorescence on mouse tissues

Mouse tissue was sectioned at 30 µm on a cryostat and stained as free floating sections using Netwell baskets [3]. Briefly, sections were washed on TBS (Tris Buffered Saline) and followed by permeabilization in TBS supplemented with 0.05% Triton X-100 (TBS-T). After washing, citric acid-based antigen retrieval for 20 min at 94°C was performed. Following antigen retrieval, sections were cooled to room temperature (RT), washed 3 times in TBS-T, and incubated in blocking buffer (TBS-T supplemented with 5% normal donkey serum and 1% bovine serum albumin [BSA]) at RT for 2 h. Sections were then incubated in primary antibody solutions diluted in blocking buffer for 48 h at 4 °C. After 48 h, sections were washed 3 times in TBS-T and incubated with fluorescent secondary antibodies diluted 1:500 in blocking buffer for 2 h at RT. After incubation in secondary antibodies, the sections were washed 2 times in TBS-T, followed by a 10 min incubation in DAPI nuclear stain (1:5.000 in TBS-T), and 2 final washes in TBS. Sections were then mounted on microscope slides, Primary antibodies are described in Additional file 5: Table S2 and secondary antibodies were: Alexa Fluor 546 donkey anti-mouse and Alexa Fluor 488 donkey anti-rabbit (1:500, Life technologies). Confocal microscope Espectral Leica TCS SP5 was used to take the images. To quantify the percent of CB_2_-TAU^+^ neurons, neuron counts were performed using Fiji Software (http://fiji.sc/Fiji) in aprox. 30 neurons/animal (n = 4 animals) of the hippocampus sections. To determine the area of the dendate gyrus, a total of 3 images per condition was analysed as follows. The images are transformed into 16 bits with the Image J program. Then, with the "Free Hand Selection" tool of the Imagen J program, we manually selected only the dentate gyrus area of each image stained with DAPI. The dimension of the dentate gyrus inside the selected area was quantified using the "Measure" tool in Image J program and the raw results measured in inches were represented.

### Sarkosyl-soluble and -insoluble fractions of mouse hippocampi

Ipsilateral hippocampi were homogenized in Buffer A (0.1 M Buffer MES pH 7, 1 mM EDTA, 0.5 mM MgSO4, 1 M sucrose, 1 mM NaF, 1 mM Na3VO4, 10 µg/ml leupeptin and phenylmethylsulfonyl fluoride (PMSF)). Homogenates were centrifuged at 20.000 rpm for 20 min at 4 °C. To obtain the sarkosyl-insoluble fraction (SI), the pellets were resuspended in RAB buffer described in [58], vortexed for 1 min at room temperature, incubated at 4 °C overnight and then centrifuged at 69.000 rpm for 30 min at 4 °C. The supernatants were collected as sarkosyl-soluble fractions (SS) and the pellets, SI fractions, were resuspended in RAB buffer with 1 × SDS protein loading buffer and incubated at 95 °C for 5 min.

### Protein extracts of mouse hippocampi

Protein lysates from ipsilateral and contralateral hippocampi were homogenized in RIPA buffer (25 mM Tris–HCl pH 7.6, 150 mM NaCl, 1 mM EGTA, 1% NP-40, 1% sodium deoxycholate, 0.1% SDS, 1 mM PMSF, 1 mM Na3VO4, 1 mM NaF, 1 μg/ml leupeptine). Homogenates were centrifuged at 13.000 rpm for 15 min at 4 °C.

### Immunoblotting

25 μg of SS, SI, and protein extracts from mouse hippocampi were resolved in SDS-PAGE and transferred to Immobilon-P membranes (Millipore). These membranes were analysed by using the following primary antibodies (Additional file 5: Table S2), and appropriate peroxidase-conjugated secondary antibodies (Amersham). Proteins were detected by enhanced chemiluminescence (ECL). Images of the immunoblotting were analyzed using ImageJ, and the lane profiles were obtained in grayscale and uncalibrated optical density.

### Human tissues

Samples and data from patients included in this study were provided by the Biobank Banco de Tejidos CIEN (PT17/0015/0014), integrated in the Spanish National Biobanks Network and they were processed following standard operating procedures with the appropriate approval of the Ethics and Scientific Committees. Immediately after brain extraction, midsagittal sectioning was performed to separate the right and left hemispheres of the brain. The left hemisphere was fixed in 10% buffered formalin for at least three weeks, and the hemisphere right were sliced and these slices were quick frozen fresh at − 50 °C (in NOVEC) and were immediately placed in at − 80 °C, where they were stored.

The frozen postmortem hippocampal tissues were obtained from four control (age 43, 58, 74 and 83 years) and four AD patients (age 80, 85, 86 and 88 years, Braak stages III-IV) within less than 6 h postmortem interval, according to the standardized procedures of Banco de Tejidos de la Fundación CIEN (Madrid, Spain). These frozen samples were used for RNA and qRT-PCR analysis. The protocol used was similar to the one described in [39].

From the same patients, we obtained 5 μm paraffinized sections from the hippocampus to perform immunohistochemistry analysis. Briefly, human tissues were deparaffinized before antigen retrieval (citric acid and sodium citrate 0.1 M). Tissues were left in a blocking solution for 1 h at room temperature and incubated with primary antibodies for 48 h. Primary antibodies were prepared in Dako REAL antibody diluent (Dako Diagnostics) and are described in Additional file 5: Table S2. Samples were incubated for 2 h at room temperature with the secondary antibodies Alexa Fluor 555 donkey anti-rabbit and Alexa Fluor 488 donkey anti-mouse (1:500, Life technologies). Finally, tissues were incubated with Sudan Black (Sigma-Aldrich) to quench endogenous autofluorescence, rinsed 2 × in 70% ethanol and stained with DAPI for 15 min.

### Statistical analysis

Data are presented as mean ± SEM (Standard Error of the Mean). To determine the statistical test to be used, we employed GraphPad Instat 3, which includes the analysis of the data to normal distribution via the Kolmogorov–Smirnov test. Besides, statistical assessments of differences between groups were analysed (GraphPad Prism 5, San Diego, CA) by unpaired Student's t-tests when normal distribution and equal variances were fulfilled. Two-way or one-way ANOVA with posthoc Bonferroni or Tukey tests were also used, as appropriate.

## Results

### Overexpression of hTAU^P301S^ induces specific changes in CB_2_ expression in a mouse model of late stage tauopathy.

**Fig. 1:**
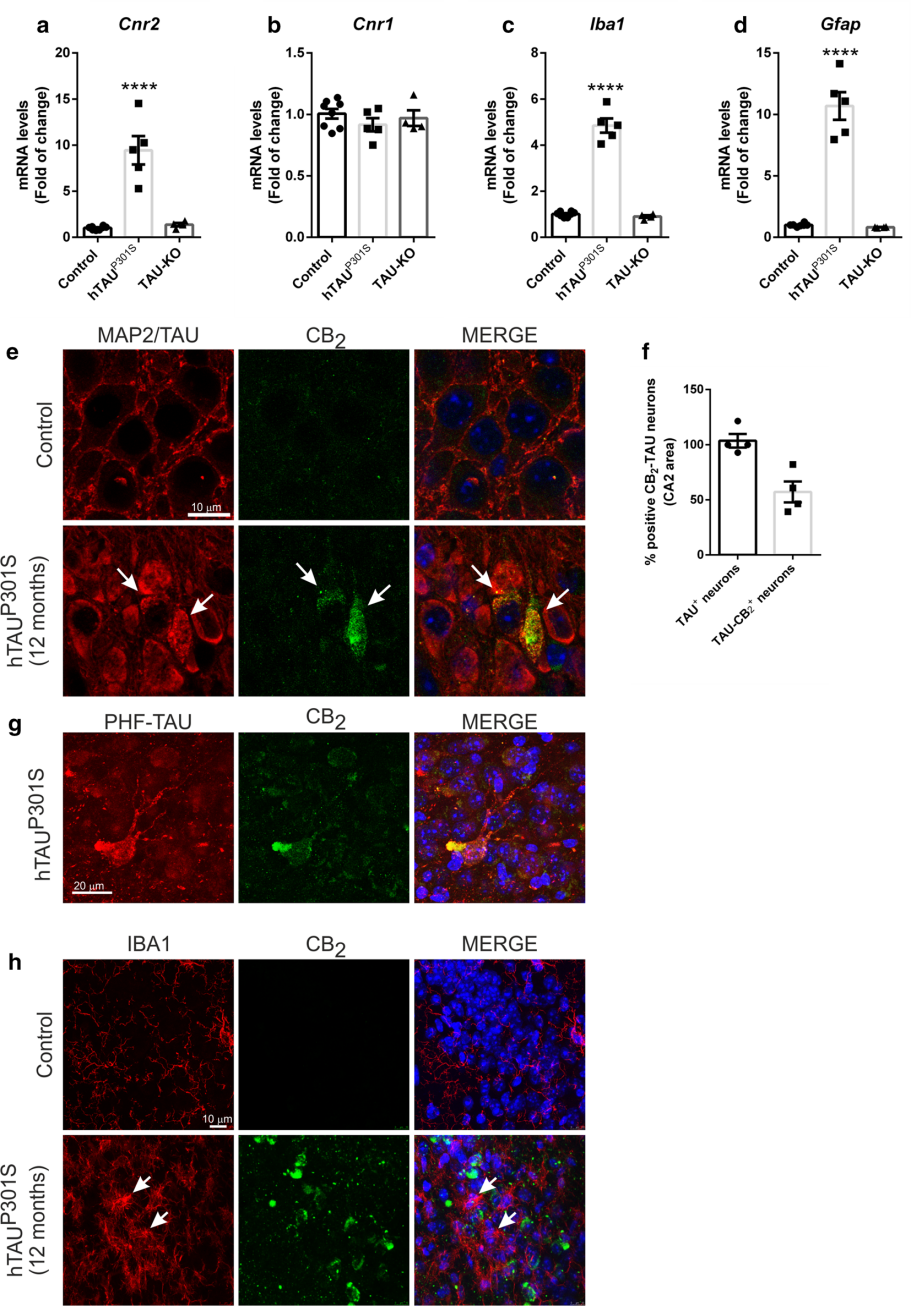
Enhanced neuronal CB_2_ expression due to hTAU^P301S^ overexpression in a late stage tauopathy transgenic mouse model. Quantitative real-time PCR determination of mRNA levels of **a** *Cnr2*, **b** *Cnr1*, **c** *Iba1*, **d** *Gfap*. All genes were normalized by *Tbp* (TATA-box binding protein) mRNA levels, n = 4–5 samples ± SEM. Asterisks denote significant differences ****p < 0.0001, comparing the indicated groups with the wild type mice according to one-way ANOVA followed by Tukey post-test. **e** Neuronal localization of the CB_2_ receptor in the hippocampus (CA2) of 12 months old hTAU^P301S^ transgenic mice. Immunofluorescence with anti-MAP2 (wild mice) or anti-TAU (transgenic mice) (red), anti-CB_2_ (green), and nuclear staining with DAPI (blue). Arrows point to hTAU^P301S^ protein aggregates. **f** Quantification of CB_2_-TAU positive neurons in the CA2 hippocampal area. Number of TAU^+^ or CB2-TAU^+^ neurons (n = 4 animals/experimental group) **g** Co-localization of neuronal CB_2_ receptor with aggregated TAU in the hippocampus (CA2). Immunofluorescence with anti-PHF-TAU (red), anti-CB_2_ (green), and nuclear staining with DAPI (blue) in the transgenic mice. **h** Immunofluorescence with anti-IBA1 (red), anti-CB2 (green), and nuclear staining with DAPI (blue)

First, we determined if the neurodegeneration induced by TAU overexpression could produce alterations in the expression levels of the CB_2_ receptor. To do this, we analysed CB_2_ mRNA levels by qRT-PCR in the hippocampus of transgenic mice that overexpressed the hTAU^P301S^ protein at 12 months of age. At this age, mice present an exacerbated neurodegenerative picture with loss of neurons in the hippocampus and neuroinflammation [68], indicating that it is a late model of tauopathy. Our results indicated that hTAU^P301S^ overexpression induced significantly *Cnr2* mRNA expression in late stages of the pathology (9.44 ± 1.54) (Fig. 1a) and this change appears to be dependent on the presence of this mutant TAU form and its potential to aggregate. In support of this dependence, TAU-deficient mice, which are perfectly viable [27, 35], did not show any alterations regarding CB_2_ receptor. Moreover, this effect was specific towards the CB_2_ receptor, as mRNA levels for *Cnr1* did not change significantly among mice from the three different genotypes (Fig. 1b). Furthermore, previous evidence from our laboratory indicated that overexpression of hTAU^P301L^ induced a neuroinflammatory process [17, 21, 39], which is a key hallmark in the neuronal degeneration in tauopathies [41]. Analysis of mRNA expression levels of *Iba1* and *Gfap*, microglial and astrocytic markers respectively, also confirmed that gliosis induced by hTAU^P301S^ overexpression (4.85 ± 0.31 and 10.68 ± 1.12, respectively) was absent in TAU-knockout mice (Fig. 1c and d). In relation with neuroinflammation, we also detected that hTAU^P301S^ overexpression significantly induced the mRNA levels of the transcription factor NF-κB (*Rela*) (1.98 ± 0.09), the master regulator of inflammation as well as the proinflammatory cytokines *Il-1β* (10.22 ± 0.64) and *Tnf* (12.98 ± 1.29) (Additional file 1: Figure S1). These results suggested that changes observed on CB_2_ expression and inflammation were directly related to hTAU^P301S^ overexpression.

Given that the elevation in the expression of the CB_2_ receptor in neurodegenerative disorders has been frequently associated with its overexpression in glial elements (e.g. reactive microglia) elicited by the local inflammatory effects [7, 14, 15], we next investigated whether the increase found in hTAU^P301S^ transgenic mice is a general effect or whether it is specific of any cell type. Therefore, we analysed specifically in which cell type CB_2_ was overexpressed using co-immunofluorescence procedures. Our data indicated that around 50% of the neurons that overexpressed hTAU^P301S^ are those that expressed the CB_2_ receptor (Fig. 1e–f). CB_2_ receptor was preferentially expressed in neurons in which the formation of aggregates of TAU protein is observed (as detected with an antibody that identifies paired helical filaments (PHF) of TAU (Thr212/Ser214) (Fig. 1g). As a control, MAP2 protein was used as a neuronal marker in the wild type mice. The finding of overexpression of the CB_2_ receptor in neurons in a neurodegenerative pathology is not frequent [59], but it is not unexpected, given the incipient evidence supporting that CB_2_ receptors may be also present in neurons in the healthy brain [44, 65]. To confirm whether this overexpression was specific or whether it also occurred in glial elements, we conducted a similar double-staining analysis, using IBA1, a marker of microglial cells. In this case, it was again observed that CB_2_ was expressed only at the neuronal level (CA2, CA3, and dentate gyrus), with no co-localization in the microglia (Fig. 1h). In turn, the difference in microglial morphology between wild-type mice (quiescent-non-reactive form) and transgenic mice (ameboid-reactive form) was strongly evident, confirming the presence of a reactive microgliosis. These data clearly indicated that hTAU^P301S^ induced selective neuronal CB_2_ expression in a late-stage tauopathy mouse model.

### Increased neuronal CB_2_ receptor expression is an early event in tauopathies.

**Fig. 2:**
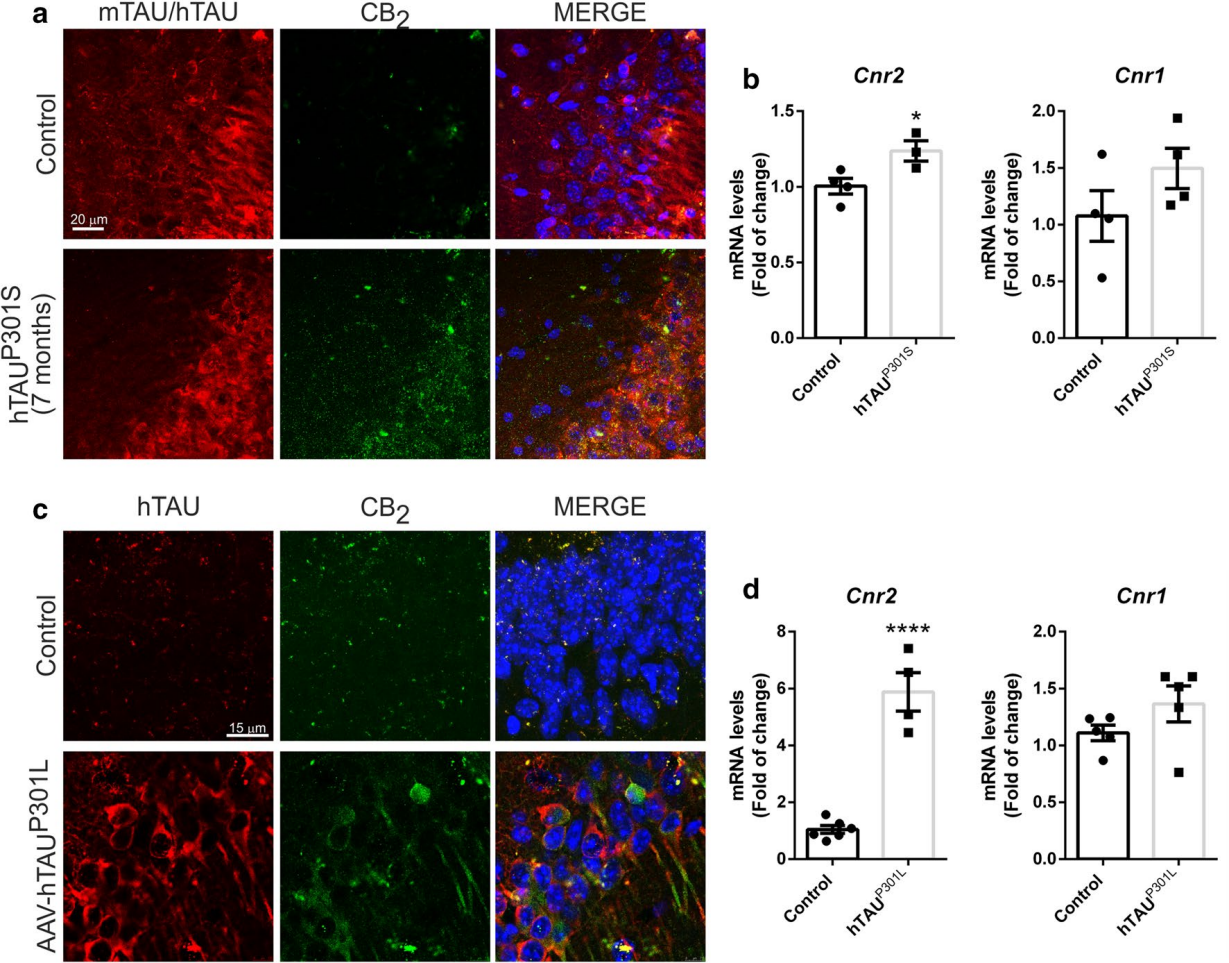
Increased CB_2_ expression is an early event in tauopathies. Analysis of 7 months old hTAU^P301S^ transgenic mice. **a** Neuronal location of the CB_2_ receptor in the hippocampus (CA2) of 7 months old hTAU^P301S^ transgenic mice. Immunofluorescence with anti-mTAU (wild type mice) or anti-hTAU (transgenic mice) (red), anti-CB_2_ (green), and nuclear staining with DAPI (blue). **b** Quantification of *Cnr2* and *Cnr1* mRNA levels, n = 4 samples ± SEM. The data has been processed with Student's t-test analysis to determine the significance of the changes. The asterisks represent the difference in significance * *p* < 0.05. **c** Neuronal location of the CB_2_ receptor in the hippocampus (CA2) in the AAV-hTAU^P301L^ mouse model. Immunofluorescence with anti-hTAU (red), anti-CB_2_ (green), and nuclear staining with DAPI (blue). **d** Analysis of wild type mice injected into the ipsilateral hippocampus with the AAV-hTAU^P301L^ vector for 3 weeks. Quantification of *Cnr2* and *Cnr1* mRNA levels, n = 4–6 samples ± SEM. The data has been processed with Student's t-test analysis to determine the significance of the changes. The asterisks represent the difference in significance **** *p* < 0.0001

Our next objective was to determine if the increase in the expression of CB_2_ receptors was also seen at earlier stages in the pathology, so that it may be considered as an early event possibly contributing to the pathogenesis. In 7 months old hTAU^P301S^ transgenic mice, we detected a significant slight increased expression of CB_2_ receptors, at mRNA (1.24 ± 0.06) and protein level (Fig. 2a and b), without alterations in the mRNA expression of *Cnr1*, *RelA*, *Il-1β* and *Tnf* (Additional file 1: Figure S1). To eliminate the possibility that these changes are due to adaptations during development, we confirmed these results in another early model of tauopathy consisting of mice stereotaxically injected into the right hippocampus with an AAV-hTAU^P301L^ vector for 21 days [17, 21, 39] (Fig. 2c and d). Analysis of the samples by qRT-PCR showed that overexpression of hTAU^P301L^ induced a very significant increase in *Cnr2* mRNA levels (5.88 ± 0.67) (Fig. 2d). As in the transgenic mouse model, this effect was specific for CB_2_, since the expression of *Cnr1* did not show significant changes. These results reproduced what was observed in the hippocampus of the 12-month-old transgenic mice that overexpressed the hTAU^P301S^ protein (Figs. 1 and 2a and b), confirming that these are events that occur at an early stage of the pathology, possibly contributing to the pathogenesis. Finally, we confirmed that CB_2_ expression also takes place at the neuronal level in this additional tauopathy mouse model, using immunofluorescence for CB_2_ and TAU in hippocampus sections (Fig. 2c). As seen in Fig. 1e, there was co-localization between the CB_2_ receptor and neurons that overexpress the hTAU^P301S^ or hTAU^P301L^ protein, confirming again its expression at the neuronal level.

### AEA levels are induced by overexpression of hTAU^P301L^ in the hippocampus.

**Fig. 3:**
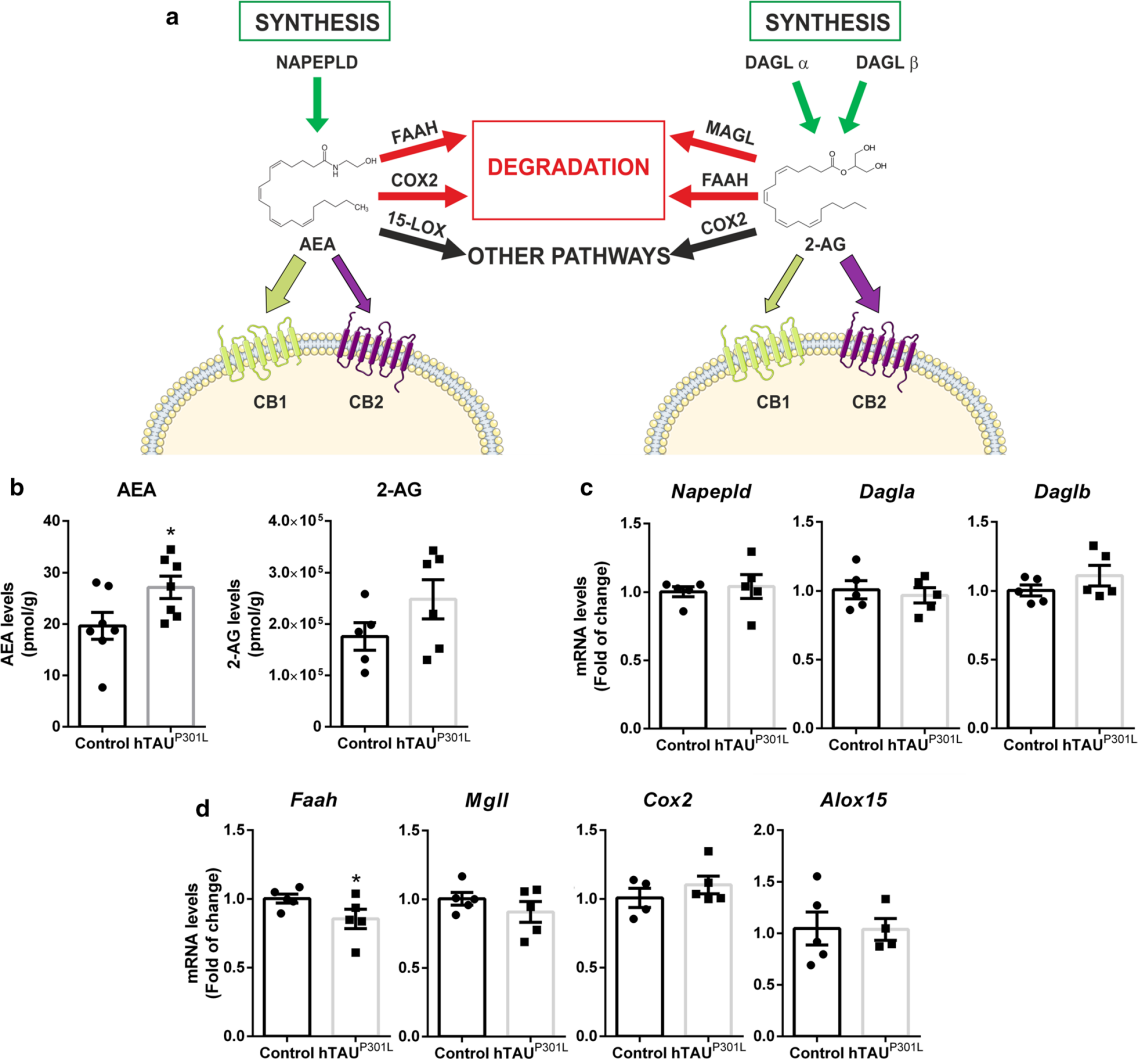
hTAU^P301L^ overexpression increased AEA levels in the hippocampus. **a** Scheme of the ECS: main receptors, ligands, and enzymes involved in the endocannabinoid signalling pathway. **b** Analysis of AEA and 2-AG levels (pmol/g) measured by LC–MS on the ipsilateral and contralateral hippocampi from wild type mice injected with the AAV-hTAU^P301L^, n = 8 samples ± SEM. **c** qRT-PCR determination of mRNA levels of *Napepld*, *Dagla*, and *Dglb*, involved in the synthesis of AEA and 2-AG, and **d** *Faah* and *Mgll*, involved in the degradation of AEA and 2-AG. *Cox2* and *Alox15*, involved in other pathways. All measures were normalized by *Tbp* mRNA levels. n = 5 samples ± SEM. Asterisks denote significant differences *p < 0.05, according to Student's t-test

Besides cannabinoid receptors, we must take into consideration that TAU overexpression may also induce changes in other elements of the endocannabinoid system. Therefore, we analysed the levels of ECs and the enzymes involved in their synthesis/inactivation in hippocampal samples of mice injected stereotaxically into the right hippocampus with an AAV-hTAU^P301L^ vector (ipsilateral). As we have previously mentioned, AEA and 2-AG are the two main ECs, with different affinities for CB_1_ and CB_2_ receptors [51] (Fig. 3a). LC–MS analyses showed that hTAU^P301L^ overexpression increased significantly the levels of AEA (Control: 19.64 ± 2.60; hTAU^P301L^: 27.13 ± 2.18) and to a lesser extent (only as a trend), those of 2-AG (Control: 175,773 ± 26,855; hTAU^P301L^: 248,234 ± 38,184) (Fig. 3b). In order to check whether these results could be due to alterations in the expression of the enzymes involved in their synthesis, we measured mRNA levels of *Napepld*, *Dagla*, and *Daglb*, but we did not observe any changes (Fig. 3c). Then, we analysed mRNA levels of *Faah*, *Mgll*, *Cox2*, and *Alox15* (Fig. 3d), enzymes involved in the degradation of ECs and other associated pathways. In this case, we found a statistically significant decrease in the expression of *Faah* (0.854 ± 0.070) that could be responsible for the increase in AEA levels, whereas a numerical trend towards a decrease could be appreciated for *Mgll* that could be related to the trend found for 2-AG levels.

### CB_2_ receptor deficiency ameliorated cognitive impairment and reduced degeneration of the granular cell layer of the dentate gyrus induced by hTAU^P301L^ overexpression.

**Fig. 4:**
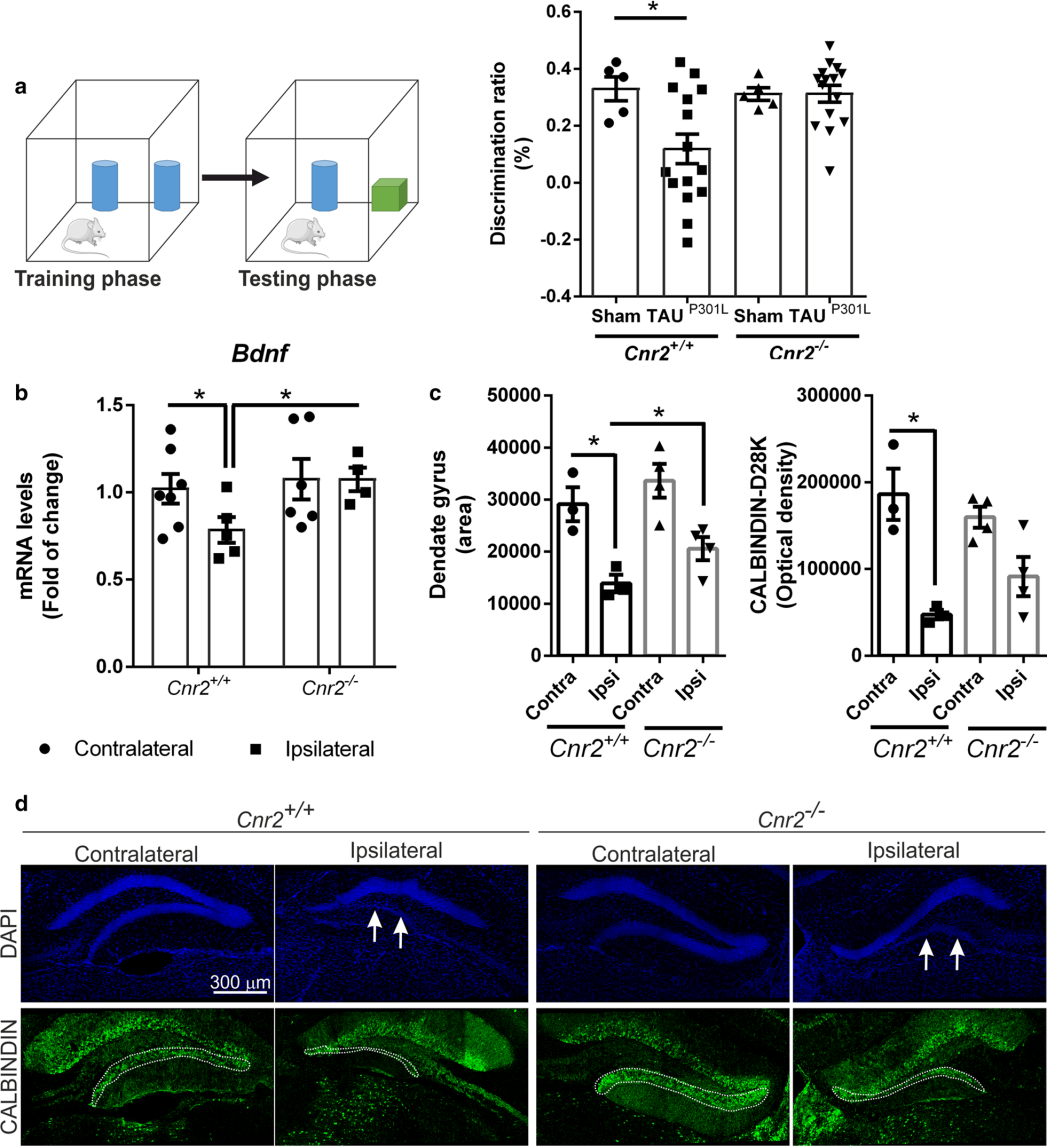
Cognitive impairment induced by hTAU^P301L^ overexpression is prevented in *Cnr2*^*−/−*^ mice. **a** Recognition memory was tested by NOR test in control mice (Sham), and *Cnr2*^+*/*+^ and *Cnr2*^*−/−*^ mice overexpressing hTAU^P301L^ (n = 8–15 per experimental group). **b** qRT-PCR determination of mRNA levels of *Bdnf*, normalized by *Tbp* mRNA levels, n = 5–7 samples ± SEM. Asterisks denote significant differences with *p < 0.05, comparing each group with the contralateral hippocampi from *Cnr2*^+*/*+^ mice or the indicated groups, according to two-way ANOVA followed by Bonferroni post-test. Reduction in the granular cell layer from the dentate gyrus due to hTAU^P301L^ overexpression is attenuated in *Cnr2*^*−/−*^ mice. **c** Quantification of the area stained with DAPI (arbitrary units) in the dentate gyrus from *Cnr2*^+*/*+^ and *Cnr2*^*−/−*^ mice injected with AAV-hTAU^P301L^. n = 3–4 samples ± SEM. Asterisks denote significant differences with *p < 0.05 comparing the ipsilateral side with the contralateral side of the indicated groups, according to two-way ANOVA followed by Bonferroni post-test. **d** Immunofluorescence staining in hippocampal sections from *Cnr2*^+*/*+^ and *Cnr2*^*−/−*^ mice injected with AAV-hTAU^P301L^ (n = 3–4 per experimental group). Blue, DAPI. Green, anti-CALBINDIN-D28K

All our results indicated that the overexpression of TAU increases the neuronal levels of CB_2_ in different tauopathy models. To determine if this increase of CB_2_ is a mechanism of the brain to fight against neurodegeneration or, on the contrary, is contributing to the pathogenesis, we overexpressed hTAU^P301L^ in CB_2_-deficient mice (AAV-hTAU^P301L^ model) and wild type animals. Then, to assess whether the CB_2_ expression affects the recognition memory alterations induced by TAU overexpression in the hippocampus, we performed the NOR test [43] two days before sacrifice. As specific controls for both genotypes, we used wild type or CB_2_-deficient mice from the same age, without hTAU^P301L^ overexpression (Sham). *Cnr2*^+*/*+^ mice injected with the AAV-hTAU^P301L^ in the hippocampus showed the expected significant decrease in the discrimination index compared to sham animals (*Cnr2*^+*/*+^ Sham: 0.329 ± 0.042; *Cnr2*^+*/*+^ -AAV-hTAU^P301L^: 0.118 ± 0.051; *Cnr2*^*−/−*^ Sham: 0.311 ± 0.022; *Cnr2*^*−/−*^ -AAV-hTAU^P301L^: 0.312 ± 0.029). However, the lack of CB_2_ receptors in *Cnr2*^*−/−*^ mice avoided this cognitive impairment caused by hTAU^P301L^ overexpression (Fig. 4a). On the other hand, the recognition memory was similar in *Cnr2*^*−/−*^*-*sham mice compared to *Cnr2*^+*/*+^*-*sham animals. These results suggested that the expression of CB_2_ could be involved in mechanisms of neuronal plasticity associated with recognition memory. We already have described that this tauopathy mouse model has alterations in synaptic plasticity [17, 21]. To assess the implication of CB_2_ in this mechanism, we determined the expression levels of Brain-Derived Neurotrophic Factor (BDNF), involved in the acquisition and consolidation of overlapping spatial memories in the dentate gyrus [49]. In *Cnr2*^+*/*+^ mice, hTAU^P301L^ overexpression significantly decreased *Bdnf* mRNA levels (0.784 ± 0.073) on the ipsilateral hippocampus (Fig. 4b). However, *Bdnf* expression levels did not change due to hTAU^P301L^ overexpression in *Cnr2*^*−/−*^ mice and remained the same as in *Cnr2*^+*/*+^-contralateral samples (Fig. 4b). These results inversely correlated with CB_2_ receptor expression levels, measured by qRT-PCR and immunofluorescence (Additional file 2: Figures S2), where higher levels of *Cnr2* (1.714 ± 0.104) correlated with lower *Bdnf* expression. In the Additional file 2: Figures S2 it can be observed that the overexpression of hTAU^P301L^ induced CB_2_ at the neuronal level, corroborating previous data (Figs. 1e and 2c), and this increase is specific since it did not occur in CB_2_-deficient mice. Thus, it seems that the increase in CB_2_ expression induced by hTAU^P301L^ overexpression in neurons is detrimental for the cognitive status.

Within the hippocampus, the area of the dentate gyrus is thought to contribute to the formation of new episodic memories [26] and the spontaneous exploration of novel environments, among other functions. Therefore, we determined if there was a correlation between the data we obtained in the NOR (Fig. 4a) and alterations in the structure of the dentate gyrus. Staining of the dentate gyrus area with DAPI, a fluorescent staining that binds strongly to adenine–thymine rich regions in DNA, indicated that hTAU^P301L^ overexpression induced the loss of part of the granular cell layer in *Cnr2*^+*/*+^ mice (Fig. 4d). Nevertheless, this loss was partially attenuated in *Cnr2*^*−/−*^ mice injected with AAV-hTAU^P301L^, as can be observed in the quantification of the area (*Cnr2*^+*/*+^-contra: 29,108 ± 3266; *Cnr2*^+*/*+^-ipsi: 13,876 ± 1670; *Cnr2*^*−/−*^-contra: 33,628 ± 3246; *Cnr2*^*−/−*^-ipsi: 20,565 ± 2222 (Fig. 4c). Concerning synaptic plasticity, we analysed the expression levels of CALBINDIN-D28K, a member of the calcium-binding protein superfamily whose expression strongly correlates with protection against TAU neurodegeneration [21]. Immunofluorescence analysis of the dentate gyrus showed that hTAU^P301L^ overexpression reduced CALBINDIN-D28K expression levels in the granular layer in *Cnr2*^+*/*+^ mice (Fig. 4d) and to a lesser extent in *Cnr2*^*−/−*^ mice (*Cnr2*^+*/*+^-contra: 186,160 ± 29,602; *Cnr2*^+*/*+^-ipsi: 47,521 ± 5462; *Cnr2*^*−/−*^-contra: 159,640 ± 12,124; *Cnr2*^*−/−*^-ipsi: 91,311 ± 22,406). Taken together, these results suggest that the lack of CB_2_ receptor protects against TAU induced neurodegeneration.

### p-TAU insolubility is reduced in CB_2_-deficient mice

In tauopathies, abnormal metabolism of TAU protein leads to its intracellular accumulation, hyper- or aberrant phosphorylation and formation of neurofibrillary tangles, which can lead to protein toxicity, cell death and neurodegeneration [33]. We analysed if the levels of soluble (SS) and insoluble (SI) TAU fractions depend on CB_2_ expression, using sarkosyl extraction as a standard protocol for investigating insoluble TAU aggregates in the brain [58].

**Fig. 5:**
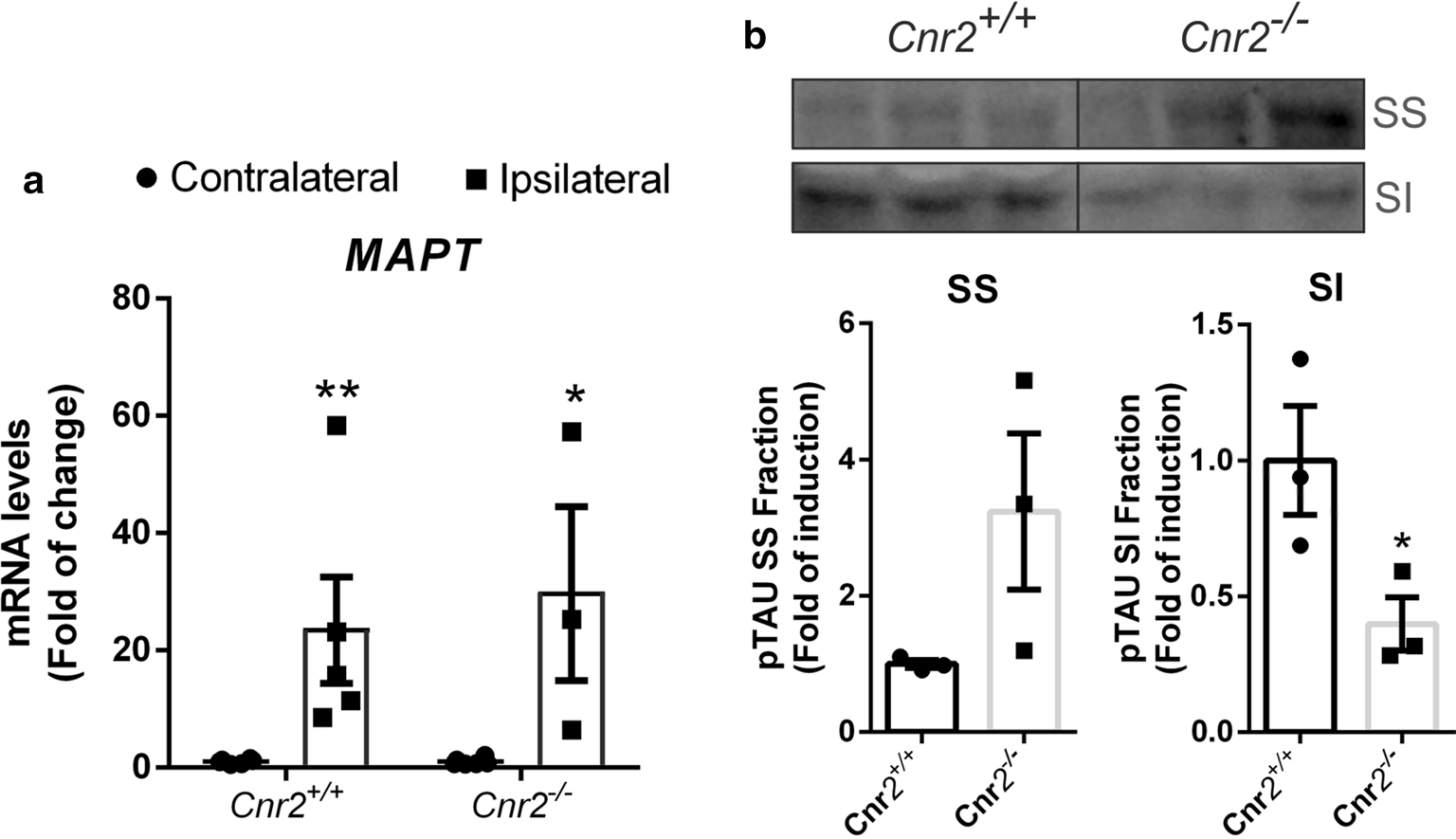
TAU-SI levels are reduced in *Cnr2*^*−/−*^ mice. **a** qRT-PCR determination of mRNA levels of *MAPT*. **b** Ipsilateral hippocampal tissue obtained from *Cnr2*^+*/*+^ and *Cnr2*^*−/−*^ AAV-hTAU^P301L^ injected mice were separated into SS and SI fractions. Levels of p-TAU-AT8 were analysed in by immunoblotting and their respective protein quantifications. n = 3 samples per experimental group ± SEM. Asterisks show significant differences with **p < 0.01 comparing each group according to a Student’s t-test

First, we determined that both genotypes expressed similar levels of *MAPT* mRNA in the injected side (Fig. 5a). It has been described that sarkosyl-insoluble (SI) TAU correlates with the pathological features of tauopathy. In the ipsilateral hippocampi, the *Cnr2*^*−/−*^ mice showed decreased hyperphosphorylated TAU at Ser202/Thr205 in the sarkosyl-insoluble fraction (SI) in comparison to *Cnr2*^+*/*+^ mice (Fig. 5b). Regarding the soluble fraction SS, we observed the opposite effect. Our data suggest that the absence of CB_2_ receptor modulates TAU levels by increasing the soluble fraction (SS), indicating a reduction of p-TAU aggregates.

### The absence of CB_2_ receptor does not change the inflammation status when overexpressing hTAU^P301L^

As we mentioned before, along with alterations in neuronal plasticity and proteostasis, neuroinflammation is a key element in neurodegenerative processes [41]. Concerning CB_2_, previous evidence from other laboratories pointed out at the possible role of CB_2_ in modulating microglial activation in different neurodegenerative disorders such as AD [12, 16], so we explored whether the up-regulatory response experienced by CB_2_ receptors in our tauopathy model could have any relation with the inflammatory response. Our results indicated that overexpression of hTAU^P301L^ was followed by the expected proinflammatory scenario reflected by increased expression of markers of astrogliosis (*Gfap*), microgliosis (*Iba1*) and proinflammatory cytokines (*Il-1β* and *Tnf*), but this was seen at the same extent in both genotypes (Additional file 3: Figures S3). Therefore, our data support that the CB_2_ receptor does not appear to play a relevant role at the level of neuroinflammation induced by hTAU^P301L^ overexpression.

### Increased expression of neuronal CB_2_ in post-mortem tissues from AD patients

**Fig. 6:**
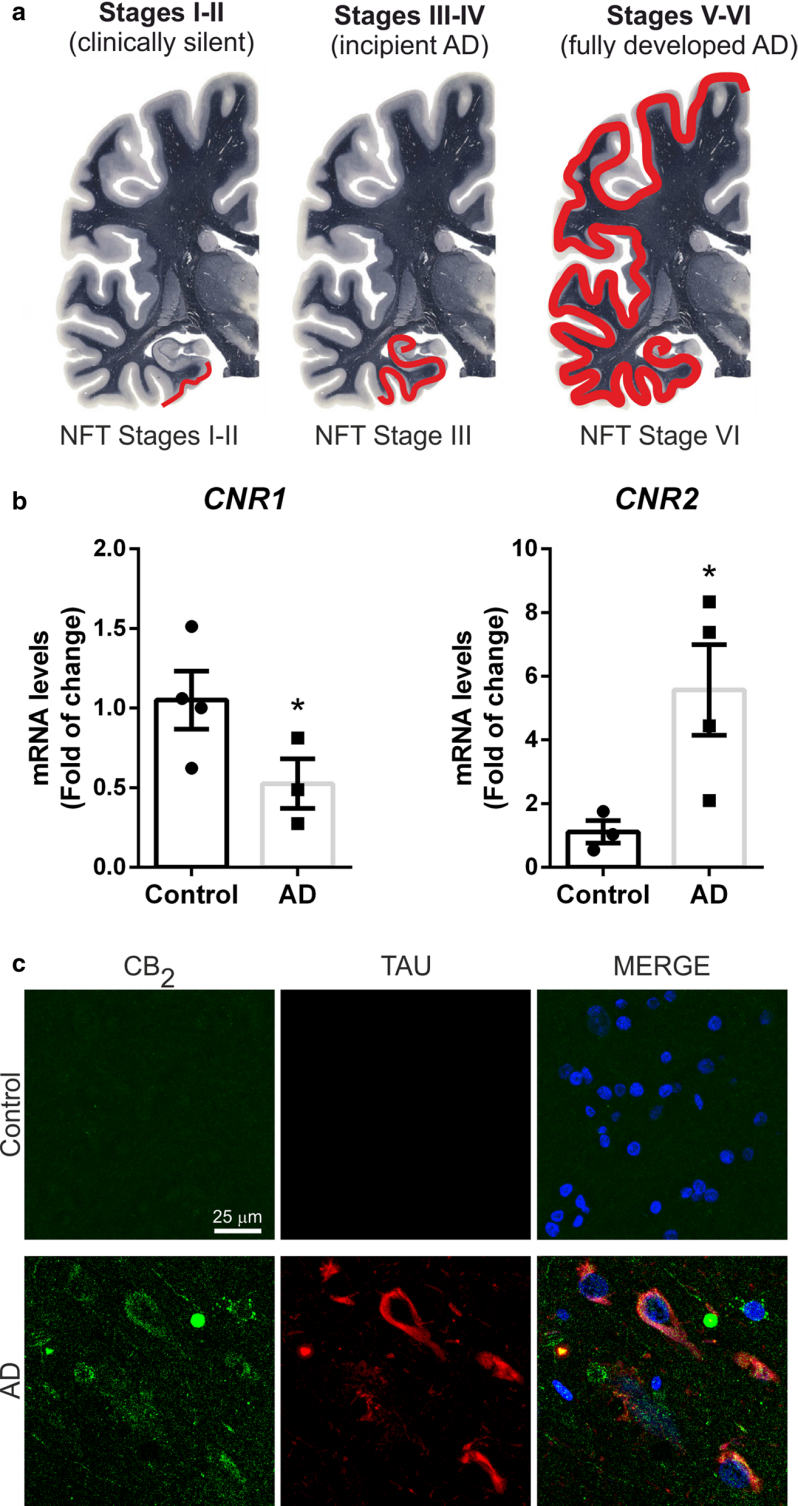
CB_2_ is increased in neurofibrillary tangle TAU positive neurons from AD patients. **a** Scheme of the different Braak stages of AD progression in the human brain: Stages I-II (transenthorinal), stages III-IV (limbic), and stages V-VI (neocortical) (Modified from http://www.thehumanbrain.info/ at the hippocampus level). **b** qRT-PCR determination of mRNA levels of CNR1 and CNR2. Both measures were normalized by TBP mRNA levels. n = 3–4 ± SEM. Asterisks denote significant differences *p < 0.05, comparing the AD patients with the control condition according to Student's t-test. **c** Double immunofluorescence staining of 15 μm-thick sections of hippocampal tissue from control (n = 4) and AD (n = 4) patients. Green, anti-CB2. Red, anti-TAU-HT7. Blue, DAPI

Our preclinical data indicated that overexpression of TAU^P301L^ induces an increase in the expression of neuronal CB_2_ and that this induction was toxic to the neuron in animal models. To determine that aberrant TAU aggregation (formation of neurofibrillary tangles (NFT)) in patients lead to similar results, in our last objective, we wanted to work with tissues from patients with tauopathies. We assume that tauopathies are an heterogenous group of pathologies and that including TAU-dependent cases of the other pathologies (e.g. TAU-dependent frontotemporal dementia) would have been desirable, but, due to its low incidence, it is difficult to collect a sufficient number of cases of this tauopathy, so we finally worked with AD, the more frequent tauopathy. We used samples from AD patients with Braak stages III-IV, when they present symptoms of incipient AD and the entorhinal and transentorhinal layers are affected (Fig. 6a) to determine CB_2_ status. Analysis of mRNA levels from both cannabinoid receptors by qRT-PCR showed that *CNR2* expression was significantly increased in AD patients (5.567 ± 1.422) compared to controls whereas *CNR1* levels were 50% decreased (0.525 ± 0.155) (Fig. 6b), indicating that the ECS is deregulated in AD, as described before [20].

Next, we explored the cell substrates in which the up-regulatory response of CB_2_ receptors takes place, so we analysed the location of CB_2_ receptors in AD, using immunofluorescence assays. Interestingly, we observed overexpression of CB_2_ receptors in those neurons that presented TAU neurofibrillary tangles (Fig. 6c), corroborating the results obtained in the murine models. These data confirmed that aberrant TAU accumulation induces CB_2_ receptor expression in human patients as in tauopathy mouse models.

## Discussion

Aberrant TAU plays a role in the pathogenesis of a variety of neurodegenerative diseases, and the study of the implication of TAU in those pathologies suggested the involvement of different molecular mechanisms. Among them, our results demonstrate for the first time that TAU overexpression increased levels of CB_2_ receptors at neuronal level and that CB_2_-deficiency protects against TAU harmful mechanism.

Although there is no specific study regarding the interaction between CB_2_ and TAU, several studies in AD models indicate a controversial role of CB_2_ in the progression of the disease. In a triple transgenic mouse model (mutations in the APP, presenilin 1 (PSEN1) and TAU genes), the deletion of CB_2_ induces AD-like TAU pathology and memory impairment [67]. Similar results were observed in another transgenic AD APP-based mouse model, in which CB_2_ receptor deficiency increased amyloid pathology and altered TAU processing [36]. Other studies indicate that in the APP/PSEN1 AD mouse model, the lack of CB_2_ receptor intensifies cortical Aβ deposition and rises the levels of soluble Aβ40 [5]. However, CB_2_ receptor ablation does not affect the survival of APP/PSEN1 mice and has no impact on memory impairment or TAU hyperphosphorylation. In this AD mouse model, it has been shown that the treatment with JWH-133, a specific CB_2_ cannabinoid receptor agonist, induced cognitive improvement due to decreased microglial reactivity and reduced expression of pro-inflammatory cytokines IL-1β, IL-6, TNFα, and IFNγ [7]. However, it has been also demonstrated that CB_2_ deletion also improves cognitive and learning deficits in another transgenic APP/PSEN1/CB2^−/−^ mice [62], in which reduced neuronal loss and decreased plaque levels were observed. Interestingly, in this study, authors demonstrated that the microglia surrounding plaques showed a less activated morphology in the absence of CB_2_ receptor, being the plaques smaller and more condensed than in APP/PSEN1 mice. Altogether, these data indicate divergent effects of CB_2_ related to APP or TAU. To date, about AD, in studies relating to the CB_2_ receptor with pathology, only models based exclusively on overexpression of the APP protein have been used, and no studies have been carried out on models based on the TAU protein, so our results are extremely novel and innovative. In this context, our results regarding the involvement of CB_2_ receptor in neurodegenerative processes that only implicate TAU protein shed light on the molecular mechanisms involved. Besides, it must be taken into account that the possible beneficial effects that have been seen with the activation of the CB_2_ receptor in these AD models are mainly based on its anti-inflammatory effects related to its expression in the microglia [4, 66], which may indicate that the direction in which the CB_2_ receptor should be modulated may depend on the type of neurotoxic event to be controlled: activation against inflammatory events in APP-based models and inhibition against neuronal deterioration in TAU-dependent models. If this is so, this should be necessarily taken into consideration when these treatments progress towards the clinical scenario given the complexity of neurodegenerative events in AD. In relation to this personalized medicine, it is also interesting to highlight the implication that modulation of the CB_2_ receptor would have in the development of disease-modifying drugs for frontotemporal dementia (FTD) spectrum disorders, although to date no change in CB_2_ levels (neither in microglia nor in neurons) has been described in this pathology, so it would be interesting to determine the status of CB_2_ and its cellular location. FTLD are classified according to the predominant protein that accumulates abnormally in cells and 40% of all FTLD are associated with aberrant TAU accumulation [52]. Therefore, the pharmacological modulation of CB_2_ as a therapeutic strategy for FTLD should be personalized, based on the molecular alterations displayed by the different type of patients.

In our study, we have shown that the neuronal induction of CB_2_ levels by overexpression of TAU is an event that is maintained throughout the neurodegenerative process until late stages of the disease and that it is dependent on TAU aggregation, since it is not altered in TAU-knockout mice. P301L or P301S mutations in the TAU protein prevent the anchoring of TAU to the microtubules and favor the aggregation of the protein, which happens, although by other mechanisms, in AD [19]. Thus, in both scenarios we have a neurotoxic form of the TAU protein, and in both cases the expression of CB_2_ was induced at the neuronal level.

Our results indicated that overexpression of hTAU^P301L^ increases AEA levels possibly by decreasing the expression of FAAH, an enzyme involved in its degradation. It has been reported that pharmacological elevation of anandamide impairs short-term memory in the hippocampus [31], indicating that in our AAV-hTAU^P301L^ mouse model the increase in AEA is implicated in cognitive impairment. These results are the first evidence of alterations in the ECS in a tauopathy mouse model. Nevertheless, it remains to be determined if variations in the levels of ECs would also be related to changes in the protein levels of these enzymes and their enzymatic activity, something already described for other neurodegenerative disorders [24].

To determine whether the increase in CB_2_ levels is beneficial or toxic to the neuron, we analysed the effect of hTAU^P301L^ overexpression in CB_2_-deficient mice. The results clearly indicate that the absence of CB_2_ improves the cognitive impairment and the synaptic plasticity (Fig. 4) induced by hTAU^P301L^ overexpression. Similar observations were found in a study of CB_2_ receptors in cerebral malaria, where the benefits were reached by blocking the receptor or ablating its gene expression [2]. However, this contrasts with most of the literature on CB_2_ receptor in other neurodegenerative/neuroinflammatory disorders, in which the benefits were reached after the activation of this receptor [6, 14, 25, 28, 32, 50]. The difference compared with our current study may be in the cell type where the CB_2_ receptor is located: glial *versus* neuronal location. Although classical expression of CB_2_ has always been related to microglia and neuroinflammatory processes [16], our results suggest that in relation to TAU, CB_2_ does not play an essential role in inflammation. Moreover, we observed that in all the tauopathy mouse models used and in AD postmortem brain samples increased CB_2_ expression occurs at neuronal levels. This agrees with recent evidence that indicates that the CB_2_ receptor can also be expressed at the neuronal level [44, 65], being involved in neuroplasticity processes like learning and memory [34], by being able to induce hyperpolarization in hippocampal neurons [55]. In line with our results, it has been demonstrated that CB_2_ disruption enhanced spatial working memory, while their overexpression reduced anxiety levels [44]. Related to the participation of the CB_2_ receptor in regulating memory, one of the main characteristics presented by patients with Alzheimer's disease (AD) is memory loss. In this work, we have confirmed an increase in *CNR2* expression in samples from AD patients. Increased CB_2_ protein expression is present in the TAU damaged neuron, with total co-localization. These results indicate that CB_2_ overexpression could be detrimental for neuroplasticity and neuronal survival and be associated with disease progression. All these pieces of evidence indicate that CB_2_ expression has different roles depending on the cell-type where it is expressed in the mature hippocampus and is important in regulating memory. Taken together, it could be speculated that when the CB_2_ receptor is expressed in the microglia, its activation has anti-inflammatory and beneficial effects against neurodegeneration, but when CB_2_ receptor is overexpressed at the neuronal level, its functionality changes radically, having a detrimental effect against neurodegeneration.

Regarding the mechanism by which the absence of CB_2_ prevents TAU toxicity, our data suggest that it could be due to an improvement in the solubility of TAU (CB_2_ deficiency led to impairment of TAU aggregate formation). It is commonly accepted that pathological features of tauopathy correlate with the levels of insoluble TAU aggregates (SI) in the brain. We found that *Cnr2*^*−/−*^ mice had less hyperphosphorylated TAU aggregates than *Cnr2*^+*/*+^ mice in the ipsilateral side of hTAU^P301L^ overexpression in the hippocampus, although they had similar *MAPT* expression levels. It has been described that CB_2_ receptors have been shown to regulate a plethora of kinases, including PI3K/AKT/GSK-3, JNK and p38 [22], which are linked to TAU phosphorylation [47, 54, 70]. These results suggested that the lack of CB_2_ could be implicated in reducing TAU phosphorylation. Further experiments will be needed to determine the involvement of these kinases in the removal of TAU aggregates and the involvement of the CB2 receptor in these processes.

## Conclusions

This study describes for the first time how TAU overexpression increases CB_2_ receptor expression at the neuronal level in the hippocampus, being an early event in tauopathies. Unlike CB_2_ induction in microglia, this neuronal CB_2_ overexpression enhances the neurodegenerative process associated with the TAU protein. This study paves the way to propose CB_2_ antagonists (or negative allosteric modulators) as an entirely novel therapeutic approach in tauopathies.

## Supplementary Information

**Additional file 1. Fig. S1**: hTAU^P301S^ overexpression in 12 months old transgenic mice induced inflammatory response in the hippocampus. (A) Analysis of mRNA levels of inflammatory genes RelA, Tnf and Il-1*β* in 12 months old transgenic mice. (B) Analysis of mRNA levels of inflammatory genes RelA, Tnf and Il-1*β* in 7 months old transgenic mice. All genes were normalized by Tbp (TATA-box binding protein) mRNA levels, n=4-5 samples ± SEM. The data has been processed with Student's t-test analysis to determine the significance of the changes. The asterisks represent the difference in significance **** p <0.0001

**Additional file 2. Fig. S2**: (A) qRT-PCR determination of mRNA levels of Cnr2. Measures were normalized by Tbp mRNA levels. n=5-7 samples ± SEM. Asterisks denote significant differences with ***p<0.001, comparing each group with the contralateral hippocampi from Cnr2^+/+^ mice or the indicated groups, according to two-way ANOVA followed by Bonferroni post-test. (B) Double immunofluorescence staining of 30 μm-thick sections of contralateral and ipsilateral hippocampus from Cnr2^+/+^ mice injected with AAV-hTAUP301L (n=3). The ipsilateral side from Cnr2^-/-^ was used as a control (n=1). Green, anti-CB2. Red, anti-TAU-HT7. Blue, DAPI.

**Additional file 3. Fig. S3**: The deficiency in CB2 does not produce changes in the neuroinflammation associated with hTAUP301L. qRT-PCR determination of mRNA levels of (A) Gfap, (B) Iba1, (C) Il-1*β*, and (D) Tnf. All genes were normalized by Tbp mRNA levels. n=5-7 samples ± SEM. Asterisks denote significant differences with *p<0.05, **p<0.01, and ***p<0.001, comparing each group with the contralateral hippocampi from Cnr2^+/+^ mice or the indicated groups, according to two-way ANOVA followed by Bonferroni post-test.

**Additional file 4. Table S1**

**Additional file 5. Table S2**

## Data Availability

The datasets analyzed during the present study are available from the corresponding author on reasonable request.

## Abbreviations

AAV vector: Adeno-associated viral
Aβ: Amyloid-β
AD: Alzheimer’s disease
AEA, or anandamide: N-arachidonoyl ethanolamine
2-AG: 2-Arachidonoylglycerol
BDNF: Brain-Derived Neurotrophic Factor
COX-2: Cyclooxygenase-2
DAG: Diacylglycerol
DAGL α/β: Diacylglycerol lipases α and β
ECs: Endocannabinoids
ECS: Endocannabinoid system
FAAH: Fatty acid amide hydrolase
FTD: Frontotemporal dementia
FTLD-TAU: Frontotemporal lobar degeneration
MAGL: Monoacylglycerol lipase
NAPE: N-arachidonoyl phosphatidylethanolamine
NOR: Novel object recognition test
15-LOX: 15-Lipoxygenase
SI: Sarkosyl-insoluble fraction
SS: Sarkosyl-soluble fractions
PMSF: Phenylmethylsulfonyl fluoride
PSEN1: Presenilin 1
