# Supplementary figures and images for: Cannabinoid receptor CB2 ablation protects against TAU induced neurodegeneration
Source: Acta Neuropathol Commun. 2021 May 17;9:90. doi: 10.1186/s40478-021-01196-5 (PMC8130522; doi:10.1186/s40478-021-01196-5)

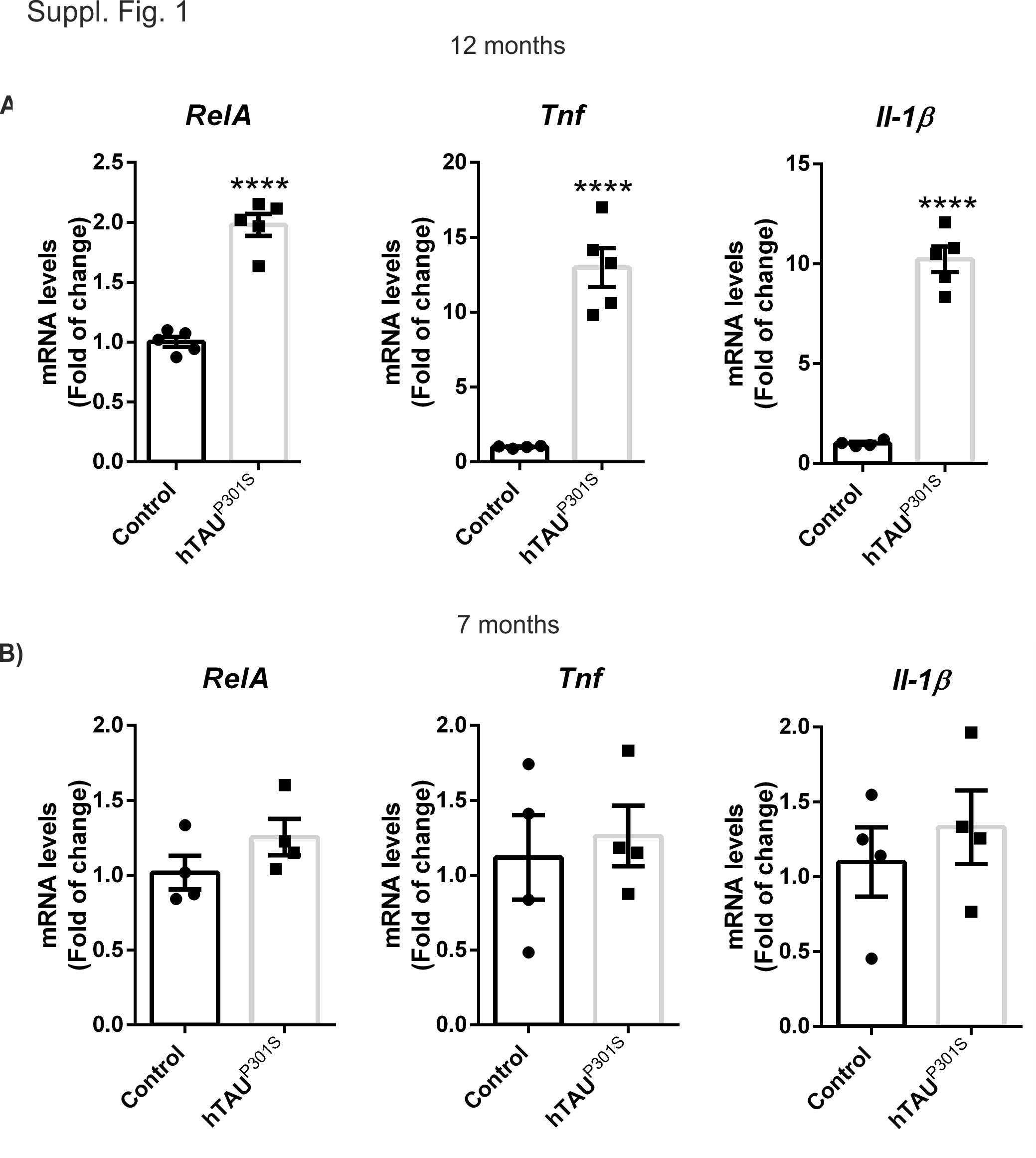

Supplement: Supplementary file 1 — Additional file 1. Fig. S1: hTAUP301S overexpression in 12 months old transgenic mice induced inflammatory response in the hippocampus. (A) Analysis of mRNA levels of inflammatory genes RelA, Tnf and Il-1β in 12 months old transgenic mice. (B) Analysis of mRNA levels of inflammatory genes RelA, Tnf and Il-1β in 7 months old transgenic mice. All genes were normalized by Tbp (TATA-box binding protein) mRNA levels, n=4-5 samples ± SEM. The data has been processed with Student's t-test analysis to determine the significance of the changes. The asterisks represent the difference in significance **** p <0.0001 [file 40478_2021_1196_MOESM1_ESM.tif]

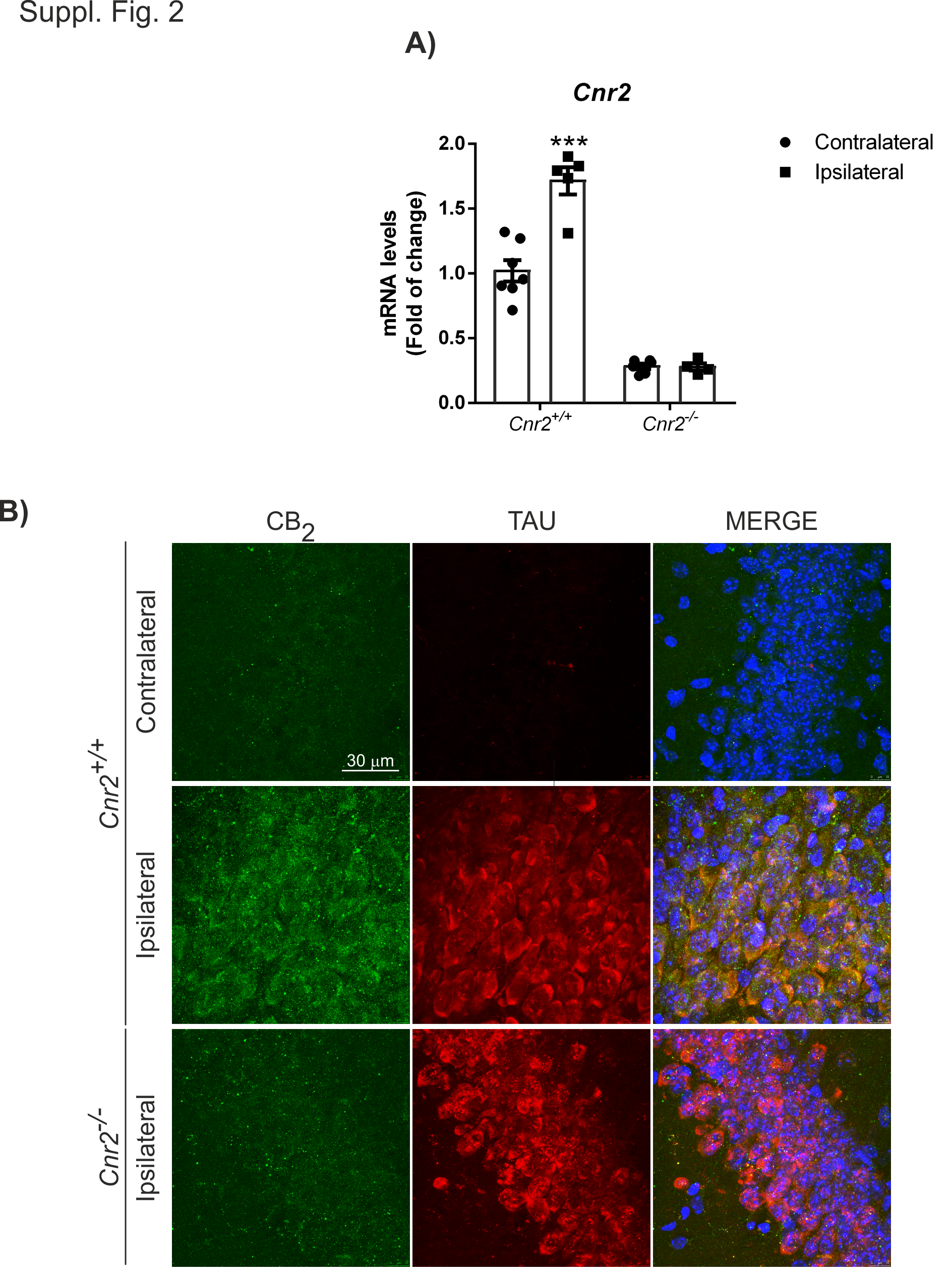

Supplement: Supplementary file 2 — Additional file 2. Fig. S2: (A) qRT-PCR determination of mRNA levels of Cnr2. Measures were normalized by Tbp mRNA levels. n=5-7 samples ± SEM. Asterisks denote significant differences with ***p<0.001, comparing each group with the contralateral hippocampi from Cnr2+/+ mice or the indicated groups, according to two-way ANOVA followed by Bonferroni post-test. (B) Double immunofluorescence staining of 30 μm-thick sections of contralateral and ipsilateral hippocampus from Cnr2+/+ mice injected with AAV-hTAUP301L (n=3). The ipsilateral side from Cnr2-/- was used as a control (n=1). Green, anti-CB2. Red, anti-TAU-HT7. Blue, DAPI. [file 40478_2021_1196_MOESM2_ESM.tif]

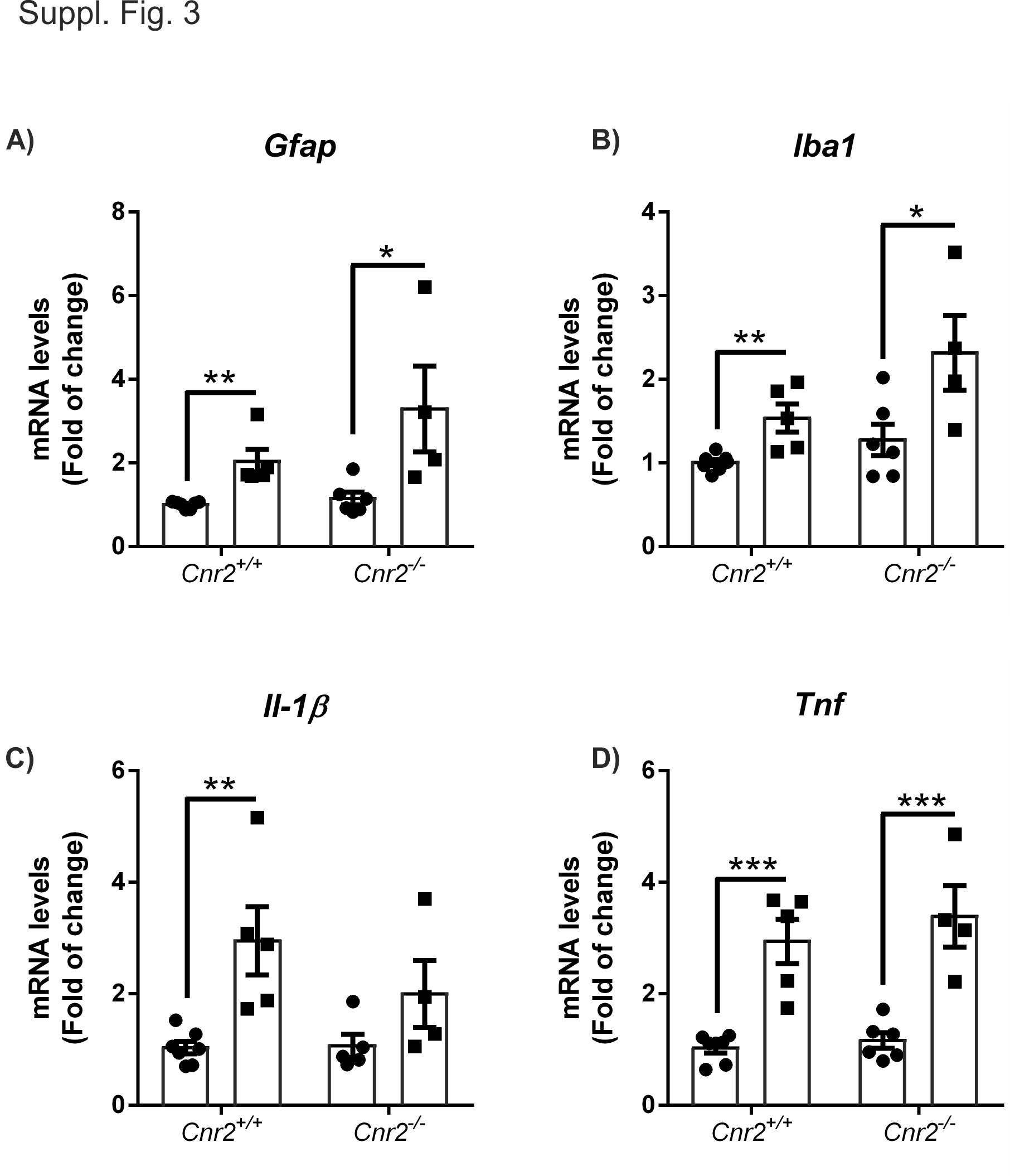

Supplement: Supplementary file 3 — Additional file 3. Fig. S3: The deficiency in CB2 does not produce changes in the neuroinflammation associated with hTAUP301L. qRT-PCR determination of mRNA levels of (A) Gfap, (B) Iba1, (C) Il-1β, and (D) Tnf. All genes were normalized by Tbp mRNA levels. n=5-7 samples ± SEM. Asterisks denote significant differences with *p<0.05, **p<0.01, and ***p<0.001, comparing each group with the contralateral hippocampi from Cnr2+/+ mice or the indicated groups, according to two-way ANOVA followed by Bonferroni post-test. [file 40478_2021_1196_MOESM3_ESM.tif]
