# Supplementary material for: Cannabinoid receptor CB2 ablation protects against TAU induced neurodegeneration
Source: Acta Neuropathol Commun. 2021 May 17;9:90. doi: 10.1186/s40478-021-01196-5 (PMC8130522; doi:10.1186/s40478-021-01196-5)
Supplement: Supplementary file 4 — Additional file 4. Table S1 [file 40478_2021_1196_MOESM4_ESM.docx]

Supplementary Table 1:

| **Gene** | **Forward primer** | **Reverse primer** |
| --- | --- | --- |
| *Alox15* | 5′ CCGCCCTCTGCCAGTGT 3′ | 5′ GCAAAATGTGTTCACTACAGCAGACT 3′ |
| *Bdnf* | 5′ GATGCCGCAAACATGTCTATGA 3′ | 5′ TAATACTGTCACACACGCTCAGCTC 3′ |
| *CNR1* | 5′ TGGGGGATATTTCGTTCTAGCG 3′ | 5′ TCGCCTGGAGTGGGAGACAT 3′ |
| *Cnr1* | 5′ TCATAGAGTCTGGGGGCAAA 3′ | 5′ CGTGACTGAGAAAGAGGTGC 3′ |
| *CNR2* | 5′ CATCACTGCCTGGCTCACT 3′ | 5′ AGCATAGTCCTCGGTCCTCA 3′ |
| *Cnr2* | 5′ TACAGAATAGCCAGGACAAGGC 3′ | 5′ ATGGATGGGCTTTGGCTTCTT 3′ |
| *Cox2* | 5′ TTCGGGAGCACAACAGAGT 3′ | 5′ TAACCGCTCAGGTGTTGCAC 3′ |
| *Dagla* | 5′ ACCCTCAAGTGCTTCGCTTA 3′ | 5′ AGCTCCGACTTGGGGATACA 3′ |
| *Daglb* | 5′ GCCTCCATCCTGAAGACCAC 3′ | 5′ CCGCTTGAGCAATTCCCTTG 3′ |
| *Faah* | 5′ AGGATTTGTTCCGCTTGGACT 3′ | 5′ AGTGGGCATGGTGTAGTTGT 3′ |
| *Gfap* | 5′ TCCTGGAACAGCAAAACAAG 3′ | 5′ CAGCCTCAGGTTGGTTTCAT 3′ |
| *Iba1* | 5′ GTCCTTGAAGCGAATGCTGG 3′ | 5′ CATTCTCAAGATGGCAGATC 3′ |
| *Il-1β* | 5′ CTGGTGTGTGACGTTCCCATTA 3′ | 5′ CCGACAGCACGAGGCTTT 3′ |
| *MAPT* | 5′ GTCCAAGTGTGGCTCAAAG 3′ | 5′ CTGGTTTATGATGGATGTTGC 3′ |
| *Mgll* | 5′ CCTGGTCAATGCAGACGGAC 3′ | 5′ AGCTCATCATAACGGCCACA 3′ |
| *Napepld* | 5′ GGGCTGTTCGGATACCTCG 3′ | 5′ CTGATAGCTTGGCGCTGGA 3′ |
| *RelA* | 5′ GGCCTCATCCACATGAACTT 3′ | 5′ CACTGTCACCTGGAAGCAGA 3′ |
| *Tbp* | 5′ TGCACAGGAGCCAAGAGTGAA 3′ | 5′ CACATCACAGCTCCCCACCA 3′ |
| *Tnf* | 5′ CATCTTCTCAAAATTCGAGTGACAA 3′ | 5′ TGGGAGTAGACAAGGTACAACCC 3′ |
