# Supplementary material for: Cannabinoid receptor CB2 ablation protects against TAU induced neurodegeneration
Source: Acta Neuropathol Commun. 2021 May 17;9:90. doi: 10.1186/s40478-021-01196-5 (PMC8130522; doi:10.1186/s40478-021-01196-5)
Supplement: Supplementary file 5 — Additional file 5. Table S2 [file 40478_2021_1196_MOESM5_ESM.docx]

Supplementary Table 2:

| **Antibody** | **Source** | **Catalog number** | **Dilution** |
| --- | --- | --- | --- |
| β-ACTIN | Santa Cruz Biotechnology | sc-1616 | 1:4.000 WB |
| CALBINDIN-D28K | Synaptic Systems | 214.002 | 1:500 IF |
| CB_2_ | Abcam | ab3561 | 1:200 IF |
| IBA1 | Wako Chemicals | 019-19741 | 1:500 (IHC) |
| MAP2 | Sigma-Aldrich | M4403 | 1:200 IF |
| p-TAU-AT8  (Ser202/Thr205) | Thermo Fisher Scientific | #MN1020 | 1:1000 IF  1:500 WB |
| PHF-TAU (AT100)  (Thr212, Ser214) | Thermo Fisher Scientific | #MN1060 | 1:200 IF |
| Human TAU total | Santa Cruz Biotechnologies  ThermoFisher Scientific | sc-5587  #MN1000B | 1:200 (IHC)  1:100 (IHC) |
| TAU, PAD, CLONE TNT-1 | MERCK | MABN471 | 1:500 |
